# Redox-Mediated Mechanism of Chemoresistance in Cancer Cells

**DOI:** 10.3390/antiox8100471

**Published:** 2019-10-10

**Authors:** Eun-Kyung Kim, MinGyeong Jang, Min-Jeong Song, Dongwoo Kim, Yosup Kim, Ho Hee Jang

**Affiliations:** 1 Department of Biochemistry, College of Medicine, Gachon University, Incheon 21999, Korea; ekkim@gachon.ac.kr (E.-K.K.); apnea4001@naver.com (M.J.); neptune6nrg@hanmail.net (M.-J.S.); 2 Department of Health Sciences and Technology, GAIHST, Gachon University, Incheon 21999, Korea; cd0575@naver.com (D.K.); youandkys@naver.com (Y.K.)

**Keywords:** reactive oxygen species, antioxidant proteins, chemoresistance, oxaliplatin, 5-Fluorouracil

## Abstract

Cellular reactive oxygen species (ROS) status is stabilized by a balance of ROS generation and elimination called redox homeostasis. ROS is increased by activation of endoplasmic reticulum stress, nicotinamide adenine dinucleotide phosphate (NADPH) oxidase family members and adenosine triphosphate (ATP) synthesis of mitochondria. Increased ROS is detoxified by superoxide dismutase, catalase, and peroxiredoxins. ROS has a role as a secondary messenger in signal transduction. Cancer cells induce fluctuations of redox homeostasis by variation of ROS regulated machinery, leading to increased tumorigenesis and chemoresistance. Redox-mediated mechanisms of chemoresistance include endoplasmic reticulum stress-mediated autophagy, increased cell cycle progression, and increased conversion to metastasis or cancer stem-like cells. This review discusses changes of the redox state in tumorigenesis and redox-mediated mechanisms involved in tolerance to chemotherapeutic drugs in cancer.

## 1. Introduction

**Figure 1:**
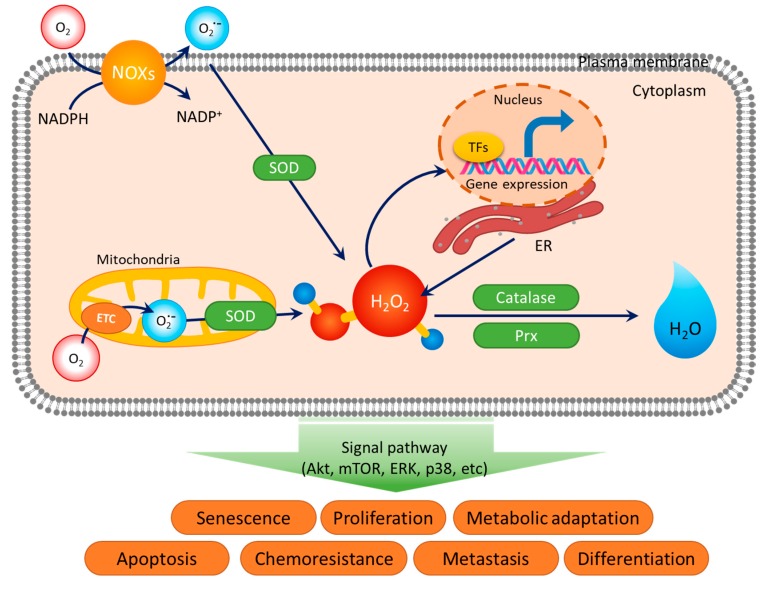
Redox homeostasis between generation and elimination of reactive oxygen species (ROS). ROS production is regulated by the nicotinamide adenine dinucleotide phosphate (NADPH) oxidases (NOXs) in membranes, the electron transport chain (ETC) of the adenosine triphosphate (ATP) synthesis process in mitochondria, and the protein synthesis process in endoplasmic reticulum (ER) during O_2_ consumption. Alternative levels of ROS induce DNA damage or transcription factors (TFs)-mediated gene expression in the nucleus. The superoxide anion (O_2_•^−^) produced intracellularly is neutralized to hydrogen peroxide (H_2_O_2_) by the superoxide dismutase (SOD) family. H_2_O_2_ are detoxified to H_2_O by catalase and peroxiredoxin (Prx). ROS regulate cellular processes such as proliferation, apoptosis, chemoresistance, and differentiation through a variety of signaling pathways.

Reactive oxygen species (ROS), as second messengers, function in various cellular signal pathways in normal cells and cancer cells [1]. Redox homeostasis is regulated by a balanced status between ROS production and scavenging (Figure 1) [1,2]. Signal cascades induced by stimuli can lead to ROS generation from ligand-receptor interactions [2,3,4]. Molecules that can directly penetrate the cell membrane, such as lipophilic growth hormones (steroid hormones and thyroid hormones) and chemical drugs, can activate mitochondrial-mediated ROS generation [5,6,7]. Although various stimuli can induce changes in ROS and affect the physiological response in cells, the antioxidant proteins stabilize ROS levels to maintain redox homeostasis [8]. Superoxide dismutase (SOD), catalase, peroxiredoxin (Prx), and nuclear factor erythroid 2-related factor 2 (Nrf2) are antioxidant modules [9]. Local ROS level, as a second messenger, amplifies only the specific region where receptor activation transduces a linear signal response. [3,10]. This process is regulated locally by ROS inducers and antioxidant modules to overcome the possibility that the alternative ROS can affect whole cells [3].

Many studies have shown that redox imbalances can induce signaling pathways that promote cancer progression, senescence, differentiation, and apoptosis [8]. Cancer cells show enhanced glycolysis-mediated metabolisms to overcome over-utilized ATP or alter cellular signal pathways [11]. Thus, many cancer cells upregulate antioxidants as protection against their high levels of ROS. Chemotherapeutic agents can induce increased ROS levels, and most cancer cells treated with chemotherapy suffer from ROS-mediated apoptosis [12]. Some cancer cells evolve mechanisms to escape ROS-mediated apoptosis and acquire tolerance to anti-cancer drugs [13]. The ROS system has a dual function that can either induce apoptosis or allow cells to adapt to various environments. ROS regulation has thus been a critical target for the development of anticancer drugs [14]. In this review, we discuss the change of redox balance by the generation or removal of ROS in tumorigenesis and redox-mediated mechanisms of the chemoresistance in chemotherapy.

## 2. Redox Homeostasis in Tumorigenesis

### 2.1. ROS Generation

Intracellular redox functions as an oncogenic factor for the activation of signal transduction in tumorigenesis [9]. ROS consists of both free radical and non-radical groups. The free radical group includes superoxide anion (O_2_•^−^), peroxyl radical (RO_2_•), hydroxyl radical (•OH), and hydroperoxyl radical (HO_2_•). Hydrogen peroxide (H_2_O_2_) and single oxygen (^1^O_2_) are classified as non-radical ROS. Production of intracellular ROS is generated by ATP synthesis in mitochondria, protein synthesis in the endoplasmic reticulum (ER), and activation of (NADPH) oxidase NOX family members [5].

#### 2.1.1. ATP Synthesis in Mitochondria

Mitochondria generate intracellular ROS during the electron transport chain (ETC) of the ATP synthesis process [15]. The homeostasis of ROS in mitochondria is maintained by antioxidant proteins. Upon electron leakage of the ETC, the abnormal ROS status of mitochondria can activate apoptosis in carcinoma cells [15,16].

Cancer cells show increased metabolism for their elevated proliferation and migration. Cancer cells have significantly increased the ATP production as well as the ROS [15,16,17,18]. Chemoresistant cancer cells require the active pump of the ATP-driven multidrug efflux, such as ATP-binding cassette (ABC) transporters [19]. The role of these transporters is to pump out intracellular toxic chemical drugs into the extracellular region by ATP hydrolysis [20]. ABC transporters include multidrug resistance-associated protein 1 (MRP1/ABCC1), breast cancer resistance protein/ABC subfamily G member 2 (BCRP/ABCG2), ABC subfamily B member 5 (ABCB5), and multidrug resistance protein 1/ABC subfamily B member 1 (MDR1/ABCB1) [19,20,21]. Enhanced ROS level is generated by the ETC, but the antioxidant machinery is also induced to adapt to the higher ROS level. Thus, regulation of the ETC in mitochondria may be a good approach to overcome chemoresistance via the blockage of routes that generate ATP or the dysregulation of ROS production that induces apoptosis.

#### 2.1.2. Endoplasmic Reticulum (ER)

The ER is a dynamical cellular organelle that plays a role the protein folding system, which regulates almost all of the membrane proteins and secretory proteins for post-translational modification [22,23]. The intracellular H_2_O_2_ in the process of protein synthesis in ER is generated by the formation of disulfide bridges during the induction of functional three-dimensional structures via protein disulfide isomerase (PDI) and other oxidoreductases [24,25]. Thus, the intracellular ROS status of ER maintains the relatively high level [26,27]. The ER-stress induced response is involved in the survival, metastasis, and angiogenesis in cancer cells under rough microenvironmental situations [28,29].

#### 2.1.3. NADPH Oxidases (NOXs)

The NOX family members consist of NOX1–5 and dual oxidase (DUOX) 1 and 2 [30]. H_2_O_2_ and O_2_•− produced by NOXs function as secondary messengers to transduce signals in response to various growth-related factors and chemical drugs [31,32,33]. NOX-induced ROS production provokes the acquisition of chemoresistance and contributes to cancer progression [34,35].

NOX1 is mainly located at the plasma membrane and endosome. NOX2 and NOX5 are located in the ER and plasma membrane [36,37]. NOX3 is localized mostly at the plasma membrane as well as mitochondria. NOX4 is also localized at the plasma membrane, the ER, the inner membrane of mitochondria and nucleus [38]. DUOX1 is located in the plasma membrane and ER, while DUOX2 is localized to the plasma membrane, ER, and cytosol as well as mitochondria and nucleus [30].

NOX1 and NOX5 regulate the drug efficacy of chemotherapy in prostate cancer [39]. NOX2 expression is related to invasion and progression in gastric cancer and acute myeloid leukemia (AML) [40,41]. NOX4 has a function of regulation in drug resistance [34]. Overexpression of DUOX and NOX4 has been detected in human thyroid tumors [35]. Due to the limits of expression in the inner ear epithelial cells and cochlea, the precise role of NOX3 in cancer is unknown [30,42]. In pancreatic ductal carcinoma, the elevated ROS level by activated NOXs induces tolerance against chemotherapy and radiation therapy [43,44,45]. The target of NOXs is a druggable strategy to treat cancer by drugs that inhibit NOXs, and cancer cells can be treated by inducing redox state-mediated triggers of apoptosis [46].

### 2.2. ROS Elimination

Redox homeostasis is regulated by the antioxidant enzymes. Cancer cells maintain sustained overexpression of antioxidant proteins to detoxify the ROS byproducts of over-activated cellular metabolisms.

#### 2.2.1. SODs

SOD is an enzyme that catalyzes the partitioning of two superoxide anions into hydrogen peroxide and molecular oxygen by the metalloenzymatic reaction [47]. SOD dependent-neutralization is important as the cell’s first barricade to ROS in the antioxidant systems. SOD has specific metal cofactors for the enzymatic activity such as SOD1 with copper (Cu) and zinc (Zn), SOD2 with manganese (Mn), and SOD3 with copper (Cu) and zinc (Zn) [48]. The SOD family is consisted of SOD1 (Cu/ZnSOD), SOD2 (MnSOD), and SOD3 (Cu/ZnSOD) [47,48]. The expression of SOD1, the most abundant SOD protein in cytoplasm, is increased in mammary carcinomas and lung carcinomas [48,49]. SOD2 was identified as downregulated in tumors in early studies, and thus SOD2 was initially considered a tumor suppressor [50]. However, recent studies have shown that SOD2 exhibits tumor-type dependent function [51,52]. SOD2 levels are higher in late-stage tumors as well as in invasive and metastatic cancers [48,50,53]. SOD2 also functions in the regulation of mitochondrial integrity and function [51,53]. Thus, SOD2 plays an important role in tumor progression. SOD3, or extracellular superoxide dismutase EcSOD, is localized in the extracellular matrix and binds to the glycocalyx in cell surfaces [54]. SOD3 functions neutralize from O_2_•^−^ by the membrane-bound NOXs to H_2_O_2_ [54,55,56]. The role of SOD3 in cancer is less known.

#### 2.2.2. Catalase

Catalase is a 62 kDa enzyme and consists of four identical subunits, including an N-terminal region for catalase reaction, a beta-barrel region for three-dimensional structure, a connection region for binding heme groups, and an alpha-helix region for NADPH binding [57,58]. The major function of catalase is to metabolize high concentrations of H_2_O_2_ for the protection of ROS-induced damage in cells. The reaction mechanism of catalase occurs in two-steps using heme groups. The first reaction is a process in which the heme cofactor reacts with a single molecule of H_2_O_2_ to produce an oxidative heme group (an oxoferryl porphyrin cation radical, which reduces the return to the previous step). In the second reaction, the oxoferryl porphyrin cation radical of catalase rapidly reacts with the second molecule of H_2_O_2_ to produce oxygen byproducts and water [58].

Another role of catalase is in the regulation of the integrin pathway during proliferation or migration [58]. Overexpression of catalase has been detected in various carcinomas, such as chronic myeloid leukemia, gastric cancer, and skin cancer [59,60,61]. Anticancer drugs also increased catalase levels in oral cancer cells, bladder cancer cells, pancreatic cancer cells, and gastric cancer cells [62,63,64,65]. Catalase expression is controlled by various mechanisms. At the transcriptional level, the expression of catalase is regulated by the activity of transcription factors on the catalase promoters, mRNA stability, and epigenetic chromatin structure [57,58]. At the protein level, the expression of catalase is affected by post-translational modification such as ubiquitination and phosphorylation [57,58].

#### 2.2.3. Prxs

Prx is a thiol-specific peroxidase protein without other cofactors for detoxification of H_2_O_2_ to H_2_O. The reaction mechanism by which Prx decomposes H_2_O_2_ into H_2_O occurs through a cycle, where peroxidatic Cys (C_P_-SH) of Prx reacts with H_2_O_2_ to oxidize to sulfenic acid (C_P_-SOH) and then back to a reduced peroxidatic Cys (C_P_-SH) state with the presence of reducing equivalents [66]. Prxs maintain cellular ROS homeostasis through this catalytic cycle [66,67]. The Prx family includes Prx1 to Prx6. Prx1 and Prx2 are located in the cytosol and nucleus, and Prx3 is localized in mitochondria. Prx4 is localized to the ER, the cytosol, and secretion. Prx5 is located in the cytosol, nucleus, mitochondria, and peroxisomes [66,68]. Prx6 is located in the nucleus, cytosol, extracellular space, and lysosome [69]. Most Prx family members are overexpressed in various carcinomas and may serve as biomarkers for cancer diagnosis [70]. Prx1 functions as an oncogenic factor in tumorigenesis. Prx1 leads to reduced DNA damage and apoptosis by detoxifying ROS. Prx1 also regulates cell signaling including NF-κB, JNK, Akt, p38 activity, VEGF, and ERK pathways [66]. Therefore, overexpression of Prx1 causes aberrant cell signaling that is beneficial for cancer cells. Prx2 function is paradoxical; Prx2 not only induces activation of the ERK pathway for promotion of metastasis but also stabilizes E-cadherin for suppression of metastasis [71,72]. Prx3 is an oncogenic factor and induces carcinogenesis via tolerance to ROS [66]. Prx4, Prx5, and Prx6 promote metastasis via clearance of increased ROS level in cancers [68,69,70].

#### 2.2.4. Nrf2

**Figure 2:**
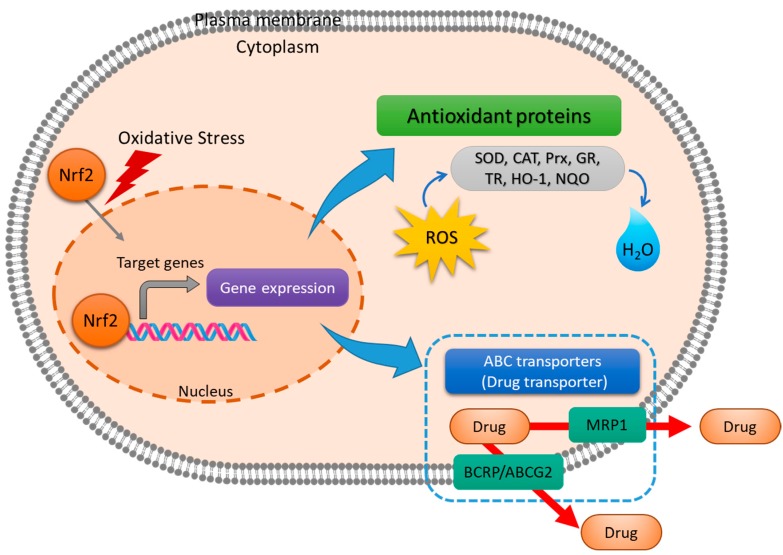
Nuclear factor erythroid 2-related factor 2 (Nrf2) regulates redox-homeostasis and chemoresistance in cells. Nrf2 induces antioxidant proteins such as superoxide dismutase (SOD), catalase (CAT), peroxiredoxin (Prx), glutathione reductase (GR), thioredoxin reductase (TR), heme oxygenase-1 (HO-1), and NAD(P)H quinone oxidoreductase 1 (NQO). Multidrug resistance protein 1 (MRP1) and breast cancer resistance protein/ATP-binding cassette subfamily G member 2 (BCRP/ABCG2) are related with drug transport and are upregulated by activation of Nrf2.

Nrf2 is a transcription factor containing the basic-region leucine zipper domain. Nrf2 is maintained at low levels by Keap1-mediated ubiquitin-dependent proteasomal degradation in the normal condition of cells [73]. Under oxidative stress or exposure to different stressors, Nrf2 is released form Keap1 due to the modification of the Keap1 Cys residue, which prevents ubiquitin-dependent proteasomal degradation of Nrf2 [73]. Nrf2 plays a role in protecting cells from oxidative stress-mediated damages through expression of target genes involved in detoxification. Nrf2-dependent gene families include antioxidant genes (SOD, CAT, Prx, GR, and TR) and genes involved in drug metabolism/transport (MRP1 and BCRP/ABCG2) (Figure 2) [74,75,76]. MRP belong to a family of membrane-anchored transporters and pump out a wide range of compounds, including peptides, lipids, organic anions, and drugs through ATP hydrolysis [76]. Carcinogenesis or chemoresistance in various cancers such as breast cancer, leukemia, neuroblastoma, and lung cancer increases the expression of Nrf2 or induces hyper-activation [77,78,79,80]. Therefore, a combination of Nrf2 inhibitors with anticancer drugs may derive therapeutic effects in patients [76].

### 2.3. Redox Homeostasis of Chemoresistance

Chemoresistance arises after long-time exposures to anticancer drugs [81,82]. The difference between temporary treatment and continuous treatment leads to different levels of ROS homeostasis in cancer cells. Cancer cells regulate ROS levels to acquire chemoresistance [13].

#### 2.3.1. Oxaliplatin Resistance

Oxaliplatin is a platinum-based drug that is widely used in lung cancer, breast cancer, pancreatic cancer, and gastric cancer [6]. The cytotoxicity mechanism of oxaliplatin involves its binding genomic DNA, which induces apoptosis in cancer cells, and the generation of ribosome biogenesis stress [6,83].

Resistance to oxaliplatin decreases the production of ROS levels [83,84,85]. However, alterations of NAPDH oxidase, ER stress, and ETC in mitochondria in oxaliplatin-resistant cells have not been deeply investigated. Oxaliplatin can also form mitochondrial DNA adducts and affect protein synthesis in mitochondria, resulting in mitochondrial abnormalities [86]. Treatment of oxaliplatin induces dysfunction in the mitochondrial respiratory chain and permeability [86,87]. High concentrations of oxaliplatin enhance ROS levels in mitochondria [86,87].

Oxaliplatin-resistant cell lines show altered expression of antioxidant proteins. Upregulated SOD1 and SOD2 detoxify drug-mediated radical species in oxaliplatin-resistant colon cancer cells [88]. SOD3 is also highly expressed in the mouse model of oxaliplatin-induced liver injury [89]. Oxaliplatin-resistant colon cancer cells have increased Nrf2 expression and exhibit chemotherapeutic effects via inhibition of Nrf2 signaling [90,91].

#### 2.3.2. 5-Fluorouracil (5-FU) Resistance

5-FU is an uracil analog drug that is widely used in various cancers. 5-FU induces DNA and RNA damage in the nucleus and mitochondria from byproducts of cellular 5-FU metabolism [92].

5-FU resistance causes a high level of intracellular ROS in colon cancer cells [93,94,95]. DUOX2 expression is altered in response to 5-FU resistance. The expression of DUOX2 is enhanced in 5-FU chemoresistant colon cancer cell lines. The upregulation of DUOX2 induces high levels of ROS and invasion ability [96]. ER-stress related factors, including PERK, GRP78, and ATF6, are upregulated in 5-FU tolerant colon cancer cells [97].

5-FU incorporation into mitochondrial DNA induces destabilization of mitochondrial DNA and protein synthesis. 5-FU can also induce ROS-mediated damage in mitochondria [98,99]. 5-FU resistance leads to a down-regulation of ATP synthesis via lower expression of ATP synthase subunits or reduced activity of ATP synthase [100,101]. Drug resistance to 5-FU induces a high level of SOD1 and Prx1 in the adaption of increased intracellular ROS conditions [102,103]. In several cancer cells, the expression or intracellular location of Nrf2 is associated with 5-FU resistance [95,103,104,105,106,107]. For example, Nrf2 is overexpressed in 5-FU resistant gastric cancer and nuclear localization, and the expression of Nrf2 is increased in 5-FU resistant colorectal cancer cells [95,105,106,107].

## 3. Redox-Mediated Mechanism of Chemoresistance

**Figure 3:**
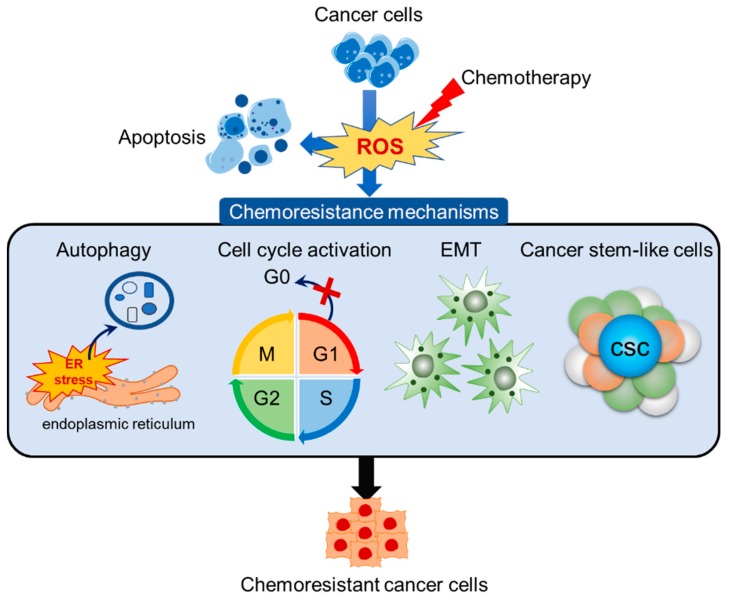
Redox homeostasis between generation and elimination of ROS. Cancer cells increase ROS-mediated apoptosis when exposed to chemotherapy treatments. Some cancer cells adapt to fluctuating ROS states through chemoresistance mechanisms. Activation of autophagy by ER stress gets rid of damaged organelles and protein aggregation. Cell cycle activation by the ignored entrance of the G0 phase increases cell proliferation in cancers. Epithelial-mesenchymal transition (EMT) enhances migration to other organs for the escapement of damaging environments. Cancer stem-like cells (CSCs) increase the expression of drug metabolic enzymes/transporters for cell survival from drug-mediated apoptosis. The specific microenvironment, called the niche, of CSCs are protected from chemotherapy. These mechanisms lead to the birth of chemoresistant cancer cells.

Multidrug resistance (MDR) is regulated by the upregulation of antioxidant proteins (hemooxygenase-1, superoxide dismutase, catalase). These antioxidant factors detoxify the altered intracellular ROS levels. Variation of the ROS level is required for chemoresistance or for the upregulation of drug efflux against chemotherapy [13,102]. ROS-mediated mechanisms for acquired chemoresistance involve ER stress and autophagy, cell cycle perturbation for overcoming cell cycle arrest, and reprogramming for promoting epithelial to mesenchymal transition or conversion to cancer stem-like cells (Figure 3) [31,104,108,109,110,111,112].

### 3.1. ER Stress-Mediated Autophagy

ROS play an important role in switching from ER stress-mediated apoptosis to autophagy in drug-resistant carcinomas [110]. Acquired resistance to anti-cancer drugs in cancer cells also results in tolerance to ER stress-mediated cell death [113]. The ER stress response also serves as a survival signal for chemoresistance in cancers [114,115]. The ER stress response is controlled by inositol-requiring enzyme-1 (IRE1), protein kinase R-like endoplasmic reticulum kinase (PERK), and activating transcription factor 6 (ATF6) in normal cells [116,117]. The loss of tumor suppressor factors or activation of oncogenes often induces activation of ER stress response-related factors to generate tumorigenesis in response to chemotherapy [97,118]. PERK is associated with the upregulation of the MDR-related protein MRP1 in chemoresistant colon adenocarcinoma cell lines [119].

Autophagy is a highly controlled degradation system of damaged organelles and protein aggregation. Autophagy is activated by starvation, organelle damage, and ER stress, and various cancers show dysfunction in autophagy [120,121]. ROS is another stimulus that can activate autophagy. H_2_O_2_ accumulation can result in oxidization of ATG4, a factor involved in the autophagic process [122,123,124]. Oxidation of ATG4 induces initiation of autophagy [125,126].

#### 3.1.1. Oxaliplatin-Resistance

Regulation of autophagy is one of the mechanisms of oxaliplatin resistance. The tolerance of colon cancer cell lines to oxaliplatin involves the down-regulation of Bcl2-modifying factor (BMF), ATG7, and Beclin-1 [127]. BMF induces necrosis, apoptosis, and autophagy [128,129,130]. Chemoresistance can be acquired by activated autophagy depending on the cell type. Oxaliplatin-resistant hepatocellular carcinoma shows autophagy activation via ROS generation and induced modulation of autophagosomes, LC3-II accumulation, and LC3 redistribution [86,131].

#### 3.1.2. 5-FU-Resistance

Chemoresistance to 5-FU is regulated by autophagy systems, although autophagy is increased or decreased in various cancers. Autophagy-related proteins, including Beclin-1, ATG5, and LC3-II, are downregulated in 5-FU-resistant human colon cancer cells [94]. However, 5-FU-resistant breast cancer cell lines show overexpression of ATG5, Beclin-1, LC3-II, and increased autophagy [132].

### 3.2. Overcoming Cell Cycle Arrest

Aberrant levels of ROS result in increased cell cycle progression to bypass arrest and acquire chemoresistance against cancer chemotherapies [108,112]. The cell cycle is regulated by positive regulators, cyclin and cyclin-dependent kinases (CDKs), and negative factors, including cyclin-dependent kinase inhibitors [108,133]. One of the hallmarks of the early stage of cancer is abnormal cell cycle progression resulting from the dysregulation of cell cycle-related factors [82,108,134].

The cell cycle progression is responsive to changes in the redox state of metabolism [112]. The redox signaling pathways alters cell cycle progression that converges as a regulator of CDK [135]. Altered ROS levels promote increased cell cycle via phosphorylation of cell cycle regulatory factors or upregulation of the cyclin family [136,137,138,139]. Cyclin D1 is overexpressed in various human carcinomas [140,141,142]. Overproduction of ROS leads to metastasis via regulation of cyclin D1, which functions in the invasion and metastatic properties of tumors [143,144].

#### 3.2.1. Oxaliplatin-Resistance

Acquired oxaliplatin resistance affects cell cycle progression. The oxaliplatin-resistant LoVo cell line has overcome oxaliplatin-mediated G2 phase arrest [145]. Overexpression of the cell cycle 5regulators cyclin D1 and B1 are reported in the oxaliplatin-resistant HT-29 cell line [146].

#### 3.2.2. 5-FU-Resistance

5-FU affects cell cycle perturbation by its incorporation in DNA and interfering with DNA synthesis [100]. Cancer cells with treatment of 5-FU have sufficient time during the cell cycle to correct the mis-incorporated fluoronucleotides and prolong DNA synthesis during the cell cycle [100,147]. However, cell lines with acquired chemoresistance show high expressions of cell cycle-related activators that result in resistance to cell cycle arrest. Breast cancer cell lines with 5-FU resistance show upregulation of cell cycle-related proteins and enhanced cellular proliferation [148,149]. 5-FU resistance results in modified expression of G1 phase cyclins in oral cancer cell lines and aberrant cell cycle regulation [100,150].

### 3.3. Epithelial-Mesenchymal Transition (EMT) and Cancer Stem-Like Cells (CSC)

The EMT process allows metastatic tumor cells to migrate to organs. Altered intracellular ROS levels may lead to the promotion of EMT in cancer cell lines that are resistant to anti-cancer drugs [81,108]. Several proteins function in the development of chemoresistance in metastatic advanced carcinomas [151,152]. Chemoresisance in cancer cells results in switching from chemotherapy-mediated apoptosis to EMT properties. Moreover, EMT-related signaling pathways, such as sonic hedgehog (SHH), Notch, TGF-ꞵ, and Wnt, overlap with renewal and maintenance of CSC [153,154]. Metastatic characterized cancer cells show phenotypes of both EMT and CSC [155,156].

Some chemoresistant cancer cell lines have characteristics of CSCs, including decreased proliferation, a greater proportion of G0/G1 cells, and increased ability of sphere-forming capacity [157,158]. The ABC transporter is related with drug resistance and cancer stem like-cells [159].

#### 3.3.1. Oxaliplatin-Resistance

Resistant to oxaliplatin results in enhanced migration and invasion abilities in colon cancer cells [160,161]. CCN2 and ID-1 are upregulated in oxaliplatin-resistant tumor cells [162,163]. CCN2 regulates cell proliferation, chemotaxis, and migration, while ID-1 is involved in blocking cell differentiation [162,163,164]. The Cx32 tumor suppressor protein is associated with positive expression of E-cadherin and negative expression of vimentin [165,166]. Cx32 level is decreased in hepatocellular carcinoma resistant to oxaliplatin [165]. Ataxin-2-line (ATXN2L) promotes migration and invasion and is elevated in oxaliplatin-resistant gastric cancer [167,168]. The cancer stem-like cell markers Oct4 and Sox2 are increased in oxaliplatin-resistant colon cancer cell lines [169].

#### 3.3.2. 5-FU-Resistance

Acquired 5-FU resistance results in altered EMT-related morphological phenotypes, such as reduced cellular adhesion, down-regulation of E-cadherin, up-regulation of N-cadherin and twist, the enlarged formation of pseudopodia and spindle-shaped morphology [170,171]. 5-FU-resistant carcinoma cell lines show high expression of vimentin, ZEB1, ZEB2, slug, snail, twist, and N-cadherin [170,172,173,174,175]. Low level of E-cadherin has been reported during 5-FU resistance of various cancer cell lines [172,174]. Increased mesenchymal factors enhanced migration. TGF-beta-mediated EMT and cancer stem-like cell capacities are reported in 5-FU-resistant pancreatic cancer cell lines [176]. CD44 is a cell surface marker of cancer stem-like cells. CD44 variant 9 is high in 5-FU-resistant gastric cancer cell lines [177,178]. Some colon cancer cells with 5-FU-resistance show features of cancer stem-like cells [179].

## 4. Conclusions

ROS are involved in physiological signal cascades in normal and cancer cells. Most of drug or growth factors induce downstream cascades that result in short-lived ROS generation. Antioxidant proteins, as ROS scavengers, play a role in the detoxification of ROS and can regulate the intensity of ROS-mediated signal transduction. Thus, cancer cells regulate the redox homeostasis to survival. ROS-mediated chemoresistance is regulated by the control of ER stress-mediated autophagy, overactivation of cell proliferation, and promotion of EMT and cancer stem-like cells.

The antioxidant system includes diverse proteins such as SOD, catalase, Prx, glutathione peroxidase, and thiol peroxidase, among others. Nrf2 has increased the expression and activity in oxaliplatin or 5-FU resistant cancer cells. Several antioxidant proteins are up-regulated in chemoresistance such as SOD1, SOD2, and SOD3 in oxaliplatin-resistant cancer cells or SOD1 and Prx1 in 5-FU chemoresistance. However, studies of variation in function or expression of other antioxidant proteins in chemoresistance are limited. Investigation of the regulation of antioxidant proteins is required for overcoming chemoresistance by regulation of the redox state, and better understanding of this process may provide new targets for the development of anti-cancer drugs.

The mechanism for acquired chemoresistance may be paradoxical. Regulation of autophagy in chemoresistance results in different responses depending on the cell type. Although chemoresistant cancer cell lines show upregulated proliferation, some chemoresistant cells become cancer stem-like cells, which are characterized by low proliferation. Thus, whether the mechanism of chemoresistance is cell type-specific should be examined in future studies. Due to the characteristic of each cell, this phenomenon (that uses different mechanisms of chemoresistance acquisition in cancer cells) is induced by signal transduction proteins such as Akt, mTOR, ERK, p38, SHH, and Wnt, depending on the activity or expression level of kinases. Most drugs change ROS status in cancers. However, ROS-mediated mechanism can occur by different pathways. Therefore, investigation of chemoresistance can reveal some of kinases with hyperactivation or hypoactivation. These results provide clues to the development of drugs in chemoresistant-related therapies.

In conclusion, clarifying the mechanisms underlying the regulation of redox-mediated chemoresistance may provide targets for drug development for overcoming chemoresistance in preclinical and clinical settings.
